# Women Are Underrepresented and Receive Differential Outcomes at ASM Journals: a Six-Year Retrospective Analysis

**DOI:** 10.1128/mBio.01680-20

**Published:** 2020-12-01

**Authors:** Ada K. Hagan, Begüm D. Topçuoğlu, Mia E. Gregory, Hazel A. Barton, Patrick D. Schloss

**Affiliations:** a Department of Microbiology and Immunology, University of Michigan, Ann Arbor, Michigan, USA; b Department of Biology, University of Akron, Akron, Ohio, USA; University of Arizona

**Keywords:** bias, gender, peer review, profession of microbiology, representation, scientific publishing

## Abstract

Despite 50% of biology Ph.D. graduates being women, the number of women that advance in academia decreases at each level (e.g., from graduate to postdoctorate to tenure track). Recently, scientific societies and publishers have begun examining internal submissions data to evaluate representation and evaluation of women in their peer review processes; however, representation and attitudes differ by scientific field, and to date, no studies have investigated academic publishing in the field of microbiology. Using manuscripts submitted between January 2012 and August 2018 to the 15 journals published by the American Society for Microbiology (ASM), we describe the representation of women at ASM journals and the outcomes of their manuscripts. Senior women authors at ASM journals were underrepresented compared to global and society estimates of microbiology researchers. Additionally, manuscripts submitted by corresponding authors that were women received more negative outcomes than those submitted by men. These negative outcomes were somewhat mediated by whether or not the corresponding author was based in the United States and by the type of institution for United States-based authors. Nonetheless, the pattern for women corresponding authors to receive more negative outcomes on their submitted manuscripts held. We conclude with suggestions to improve the representation of women and decrease structural penalties against women.

## Abstract (precis)

Barriers in science and academia have prevented women from becoming researchers and experts that are viewed as equivalent to their colleagues who are men. We evaluated the participation and success of women researchers at ASM journals to better understand their success in the field of microbiology. We found that women are underrepresented as expert scientists at ASM journals. This is, in part, due to a combination of both low submissions from senior women authors and more negative outcomes on submitted manuscripts for women compared to men.

## INTRODUCTION

Evidence has accumulated over the decades that academic research has a representation problem. While at least 50% of biology Ph.D. graduates are women, the number of women in postdoctoral positions and tenure-track positions are less than 40 and 30%, respectively (1). There have been many proposed reasons for these disparities, which include biases in training and hiring, the impact of children on career trajectories, a lack of support for primary caregivers, a lack of recognition, lower perceived competency, and lower productivity as measured by research publications (1–8). These issues do not act independently of one another; instead, they accumulate for both individuals and the community, much as advantages do (9–11). Accordingly, addressing these issues necessitates multilevel approaches from all institutions and members of the scientific community.

Scientific societies play an integral role in the formation and maintenance of scientific communities—they host conferences that provide forums for knowledge exchange, networking, and opportunities for increased visibility as a researcher. Scientific societies also frequently publish the most reputable journals in their field, facilitating the peer review process to vet new research submissions (12). Recently, scientific societies and publishers have begun examining internal submissions data to evaluate representation of and bias against women in their peer review processes. The American Geophysical Union found that while the acceptance rate of women-authored publications was greater than publications authored by men, women submitted fewer manuscripts than men and were used as reviewers only 20% of the time (13), a factor that is reported to be influenced by the gender of the editor (14). Several studies have concluded that there is no significant bias against papers authored by women (14–19). Recent reports of manuscript outcomes at publishers for ecology and evolution, physics, and chemistry journals have found that women-authored papers are less likely to have positive peer reviews and outcomes (20–23).

The representation of women scientists and gender attitudes differ by scientific field, and to date, no studies have investigated academic publishing in the field of microbiology. The American Society for Microbiology (ASM) is one of the largest life science societies, with an average membership of 41,000 since 1990. A recent statement notes that “A diverse ASM enhances the microbial sciences, increases innovation, strengthens the community and sustains the profession” and pledges to “address all members’ needs through development and assessment of programs and services” that aim to ensure “equitable access and accountability through transparent procedures and communication” (24). One of ASM’s services is the publication of microbiology research through a suite of research and review journals. Between January 2012 and August 2018, ASM published 25,818 original research papers across 15 different journals: *Antimicrobial Agents and Chemotherapy* (AAC), *Applied and Environmental Microbiology* (AEM), *Clinical and Vaccine Immunology* (CVI), *Clinical Microbiology Reviews* (CMR), *Eukaryotic Cell* (EC), *Infection and Immunity* (IAI), *Journal of Bacteriology* (JB), *Journal of Clinical Microbiology* (JCM), *Journal of Virology* (JVI), *mBio*, *Microbiology and Molecular Biology Reviews* (MMBR), *Genome Announcements* (GA, now *Microbiology Resource Announcements* [MRA]), *Molecular and Cellular Biology* (MCB), *mSphere*, and *mSystems*. Two journals, EC and CVI, were retired during the period under study and three journals, GA/MRA, MMBR, and CMR, were excluded from the analysis due to their relatively low number of submissions. The goal of our research study was to describe the population of the ASM journals both through the gender-based representation of authors, reviewers, and editors and the associated peer review outcomes.

## RESULTS

**FIG 1:**
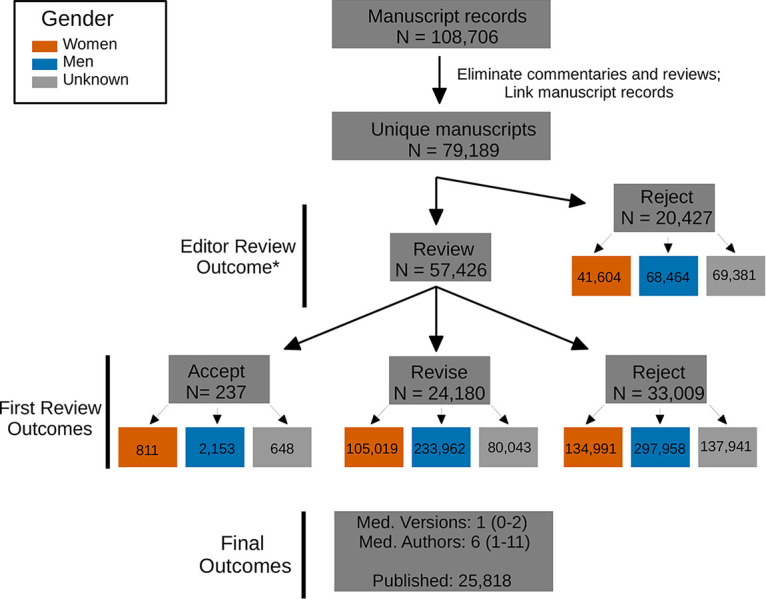
Overview of manuscript outcomes. A total of 108,706 manuscript records were obtained for the period between January 2012 and August 2018. After eliminating nonprimary research manuscripts and linking records for resubmitted manuscripts, we processed 79,189 unique manuscripts. The median number (Med.) of versions was 1 (IQR = 0 to 2) with a median of 6 (IQR = 1 to 11) authors per manuscript. As of August 2018, 34,196 of these were published at ASM journals. Revisions were requested for 24,016 manuscripts, and 53,436 manuscripts were rejected at their first submission. The number of individuals (e.g., author, editor, reviewer) involved in each category of manuscript decision are indicated in the colored boxes: women (orange), men (blue), and unknown (gray). A small number of manuscripts were given revise (242) or acceptance (1094) decisions without review (indicated by the asterisk after Editor Review Outcome).

Over 100,000 manuscript records were obtained for the period between January 2012 and August 2018 (Fig. 1). Each of these were evaluated by editors and some by reviewers, leading to multiple possible outcomes. At ASM journals, manuscripts may be immediately rejected by editors instead of being sent to peer review, often due to issues of scope or quality. These were defined as editorial rejections and identified as manuscripts rejected without review. Alternately, editors send a majority of manuscripts out for review by two or more experts in the field selected from a list of potential reviewers suggested by the authors and/or editors. Reviewers give feedback to the authors and editor, and the editor decides whether the manuscript in question should be accepted, rejected, or sent back for revision. Manuscripts with suggested revisions that are expected to take more than 30 days to address are rejected, but their authors are generally encouraged to resubmit. If the manuscripts are resubmitted, the authors are asked to note the previous manuscript and the resubmission is assigned a new manuscript number. Multiple related manuscripts were tracked together by generating a unique grouped manuscript number based on the recorded related manuscript numbers. This grouped manuscript number served dual purposes of tracking a single manuscript through multiple rejections and avoiding duplicate counts of authors for a single manuscript. After eliminating nonprimary research manuscripts and linking records for resubmitted manuscripts, we identified 79,189 unique manuscripts (Fig. 1).

We inferred the gender of both the peer review participants (e.g., editor-in-chief, editors, reviewers) and authors on the manuscripts evaluated during this time period using a social media-informed classification algorithm with stringent criteria and validation process (see Text S1 and Fig. S1 in the supplemental material). We recognize that biological sex (male/female) is not always equivalent to the gender that an individual presents as (man/woman), which is also distinct from the gender(s) that an individual may self-identify as. For the purposes of this article, we choose to focus on the presenting gender based on first names (and appearance for editors), as this information is what reviewers and editors also have available. The sensitivity, specificity, and accuracy of our method were 0.97 (maximum of 1.0) when validated against a curated set of authors (see Table S1 in the Text S1 file in the supplemental material). The accuracy was 0.99 when applied to the list of editors, whose genders were inferred by hand using Google (Text S1). In addition to identifying journal participants as men or women, this method of gender inference resulted in a category of individuals whose gender could not be reliably inferred (i.e., unknown). We included those individuals whose names did not allow a high degree of confidence for gender inference in the “unknown” category of our analysis, which is shown in many of the plots depicting representation of the population. These individuals were not included in the comparison of manuscript outcomes. Finally, we refer to editors and peer reviewers collectively as gatekeepers, which describes and recognizes their essential role in maintaining the scientific quality of manuscripts accepted (or rejected) at peer-reviewed journals (25, 26).

10.1128/mBio.01680-20.1 TEXT S1 Validation of gender inference algorithm and analysis of the impact of geographic bias. (This file contains Table S1.) Download Text S1, PDF file, 0.03 MB. Copyright © 2020 Hagan et al. 2020 Hagan et al. This content is distributed under the terms of the Creative Commons Attribution 4.0 International license.

10.1128/mBio.01680-20.2 FIG S1 (A) Equation for calculating negative bias by the genderize algorithm. C indicates a country. (B) The negative impact of each country on the overall gender inference of the full data set. The number to the right of each column is the total number of names associated with that country. Download FIG S1, TIF file, 0.3 MB. Copyright © 2020 Hagan et al. 2020 Hagan et al. This content is distributed under the terms of the Creative Commons Attribution 4.0 International license.

### Men dominated as gatekeepers and senior authors.

We first evaluated the representation of men and women who were gatekeepers during the study period. Each journal is led by an editor-in-chief (EIC) who manages journal scope and quality standards through a board of editors with field expertise that, in turn, handle the peer review process. There were 17 EICs, 17.6% of which were women. Four years before CVI was retired, the EIC of CVI transferred from a man to a woman, while JVI has had a woman as EIC since 2012. The total number of editors at all ASM journals combined over the duration of our study (senior editors and editors pooled) was 1,015, 28.8% of which were women.

**FIG 2:**
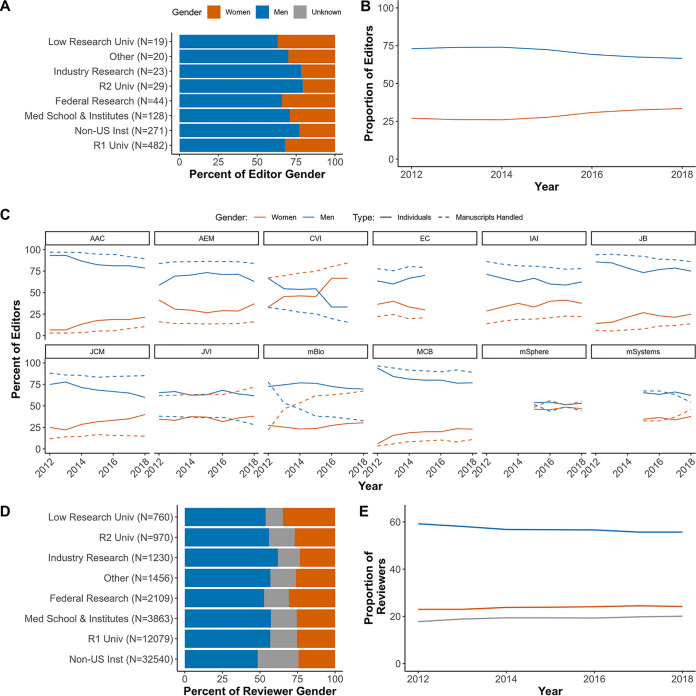
Gendered representation among gatekeepers. (A and B) Proportion of editors from institution types (A) and over time (B). Editors and senior editors are pooled together. (C) The proportion of editors (solid lines) and their workloads (dashed lines) at each of the ASM journals from 2012 to 2018. (D and E) Proportion of reviewers from institution types (D) and over time (E). (A and D) Each gender equals 100% when all institutions are summed. The total number of gatekeepers from the indicated institution are in parentheses. (B and E) Each individual was counted once per calendar year, and the proportions of both genders add to 100% per year.

Over 40% of both men and women editors were from United States-based R1 institutions, defined as doctoral-granting universities with very high research activity (27). Non-U.S. institutions and U.S. medical schools or research institutions supplied the next largest proportions of editors (Fig. 2A) (27). Since 2012, there was a slow trend toward equivalent gender representation among editors (Fig. 2B). Individual journal trends varied considerably, though most had slow trends toward parity (Fig. 2C). CVI and *mSphere* were the only ASM journals to have accomplished equivalent representation of men and women, with CVI having a greater proportion of women editors than men before it was retired. EC was the only journal with an increasing parity gap.

Altogether, 30,439 reviewers submitted reviews and 24.6% were inferred to be women. The greatest proportion of reviewers (over 50% of all groups) came from non-U.S. institutions, while R1 institutions supplied the next largest cohort of reviewers (Fig. 2D). The proportions of each gender group were consistent over time among reviewers at the ASM journals (Fig. 2E) and were representative of both the suggested reviewers at all journals combined, and the actual reviewer proportions at most journals (Fig. S2).

10.1128/mBio.01680-20.3 FIG S2 Proportion of potential reviewers at all ASM journals combined (A) and reviewers at each ASM journal (B). Download FIG S2, TIF file, 0.2 MB. Copyright © 2020 Hagan et al. 2020 Hagan et al. This content is distributed under the terms of the Creative Commons Attribution 4.0 International license.

### Editorial workloads were not proportionate.

**FIG 3:**
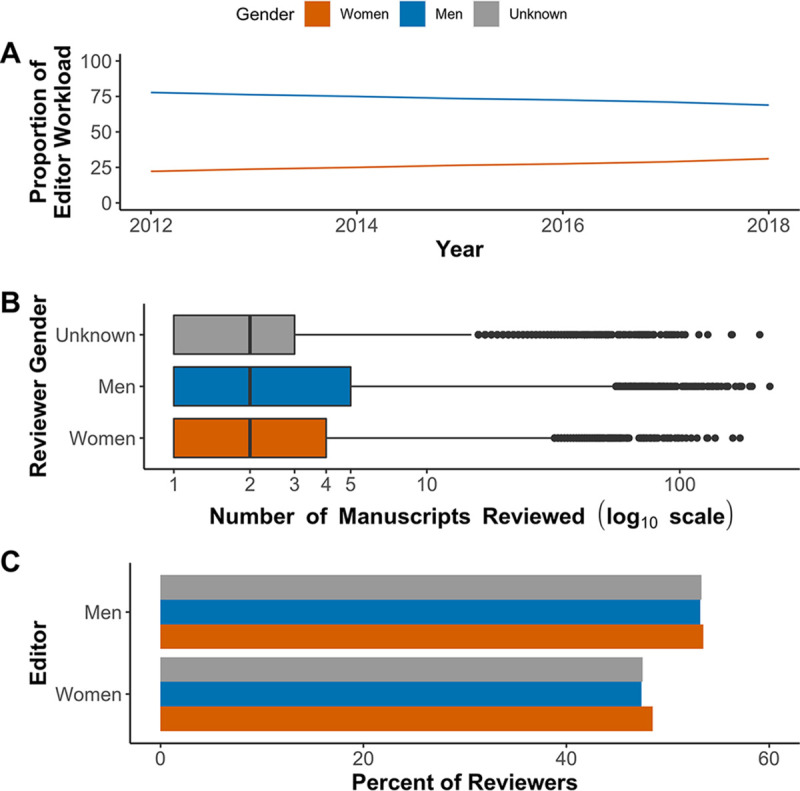
Gatekeeper workload and response to requests to review. (A) Proportion of manuscript workload handled by men and women editors, editorial rejections excluded. (B) Box plot comparison of all manuscripts by reviewer gender on a log_10_ scale. (C) The percentage of reviewers by gender that accepted the opportunity to review, split according to the editor’s gender.

To evaluate the editorial workload for each gender, we calculated the proportion of manuscripts handled by editors of each gender (excluding editorial rejections) relative to their representation. If the workload is proportionate, then the workload for each gender will be equivalent to the gender’s representation at that journal. Across all of the journals combined, men handled a slightly greater proportion of manuscripts than women relative to their respective editorial representations (Fig. 3A). This trend was present at most journals with various degrees of difference between workload and representation (Fig. 2C). For instance, at *mSphere*, both workload and representation were identical; however, CVI, *mBio*, and JVI each had periods when the workload for women editors was much higher than their representation, with corresponding decreases in the workload of men. In the years preceding its retirement, the representation of women at CVI increased, decreasing the gap in editorial workload. However, representation and relative workloads for men and women editors at JVI held steady over time, while the proportional workload for women at *mBio* has increased.

The median number of manuscripts reviewed by men, women, and unknown gendered individuals was two for each group. Half of those in the men, women, or unknown gender groups reviewed between one and five, four, or three manuscripts each, respectively (Fig. 3B). Conversely, 44.6% of men, 40.1% of women, and 48.6% of unknown gendered reviewers reviewed only one manuscript, suggesting that women were more likely than other groups to review multiple manuscripts. Reviewers of all gender groups accepted fewer requests to review from women editors (average of 47.8%) than from men (average of 53.3%; Fig. 3C). Reviewers were also less likely to respond to women editors than men (no response rate averages of 25.1 and 19.9%, respectively). Both men and women editors contacted reviewers from all three gender groups in similar proportions, with women editors contacting 76.4% of suggested reviewers and men contacting 74.1% (median of the percent contacted from each gender group).

### Women were underrepresented as authors.

**FIG 4:**
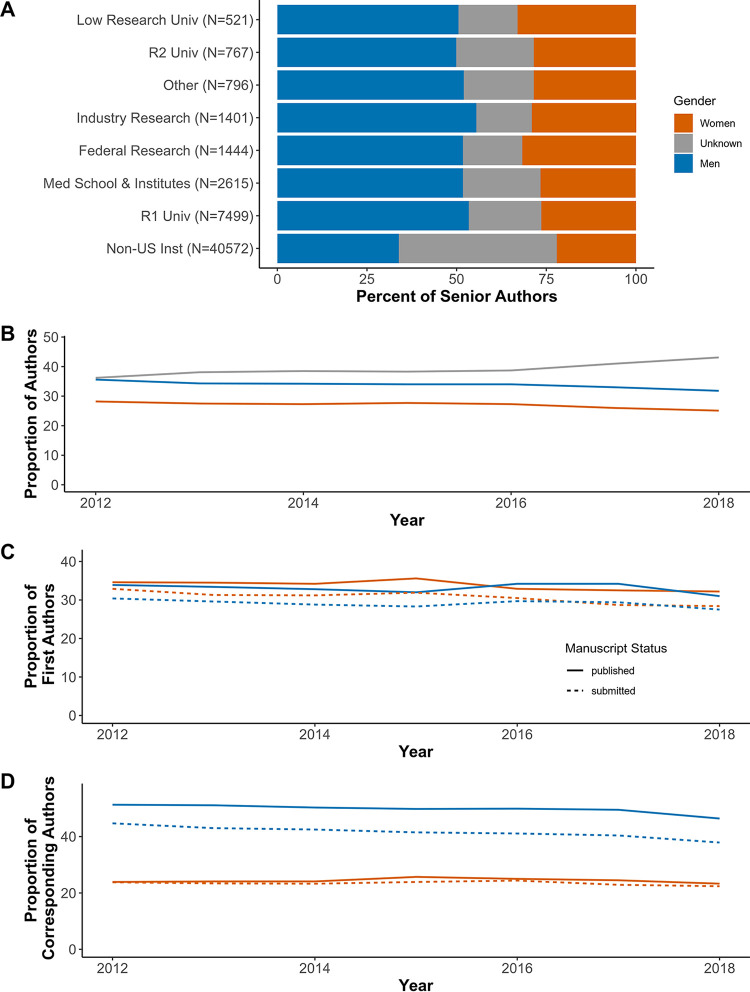
Author representation by gender. (A and B) The proportion of men, women, and unknown gender senior authors from each institution type (where the number of authors are in parentheses) (A) and men, women, and unknown (senior and co-) authors from 2012 to 2018 (B). Each individual was counted once per calendar year. (C and D) The proportion of first authors (C) and corresponding authors (D) from 2012 to 2018 on submitted manuscripts (dashed lines) and published papers (solid lines).

Globally, microbiology researchers are 60% men and 40% women (28). In September 2018, 38.4% of ASM members who reported their gender were women. We wanted to determine whether these proportions were similar for senior authors at the journals and to understand the distribution of each gender group among submitted manuscripts and published papers. We began by describing senior author (last/corresponding author) institutions by gender group. Over 60% of submitting senior authors were from non-U.S. institutions, followed by about 20% from R1 institutions. The proportion of manuscripts submitted from U.S. institutions by women senior authors was 31% versus 36% from women who were senior authors at non-U.S. institutions. Women senior authors were more highly represented at low research universities and federal research institutions than at any other U.S.-based institution (Fig. 4A). The proportions of all men and women (senior and co-) authors at the ASM journals decreased over time at equivalent rates, while the proportion of unknown gendered authors increased; the ratio of men to women authors was four to three (i.e., 57% men; Fig. 4B).

In the field of microbiology, order of authorship on a manuscript signals the type and magnitude of contributions to the finished product. First authorship and last authorship are the most prestigious. First authors are generally trainees (e.g., students or postdocs) or early career researchers responsible for performing the bulk of the project, while last authors are generally lead investigators that supplied conceptual guidance and resources to complete the project. Middle authors are generally responsible for technical analyses and methods. Any author can also be a corresponding author, which we identified as the individual responsible for communicating with publishing staff during peer review (as opposed to an author to whom readers direct questions, of which there can be multiple).

The proportion of manuscripts submitted with men or women as first authors remained constant at 29.1 and 30.7%, respectively (Fig. 4C, dashed lines). The proportions of first author published papers were nearly identical at 33.1% for men and 33.8% for women (Fig. 4C, solid lines). The proportion of submitted manuscripts with men corresponding authors remained steady at an average of 41.6%, and the proportion with women corresponding authors was 23.4% (Fig. 4D, dashed lines); the proportion of published unknown gender authors declined. Both men and women corresponding authors had a greater proportion of papers published than manuscripts submitted. Accordingly, manuscripts with corresponding authors of unknown gender were rejected at a higher rate than the rate for the manuscripts submitted. The difference between the percentage of submitted manuscripts and published papers was 8.2% when men were corresponding authors, but only 0.9% when women were corresponding authors, making their submitted and published proportions nearly equal (Fig. 4C, solid line). This trend was similar for middle and last authors (Fig. S3).

10.1128/mBio.01680-20.4 FIG S3 Proportion of all submitted (dashed line) and published (solid line) middle (A) and last (B) authors by gender at each ASM journal. Download FIG S3, TIF file, 0.2 MB. Copyright © 2020 Hagan et al. 2020 Hagan et al. This content is distributed under the terms of the Creative Commons Attribution 4.0 International license.

Of the 38,594 multiauthor manuscripts submitted by men corresponding authors, 23.5% had zero authors inferred to be women. In contrast, 7,253 (36.3%) of the manuscripts submitted by women corresponding authors had more than half of the authors inferred to be women, exceeding those submitted by men corresponding authors in both the number (3,247) and percentage (8.4) of submissions. Additionally, the proportion of women authors decreased as the number of authors increased, such that when the number of authors exceeded 30 on a manuscript (*n* = 59), the proportion of individuals inferred to be women was always below 51% (Fig. S4). Men submitted 225 single-authored manuscripts, while women submitted 69 single-authored manuscripts.

10.1128/mBio.01680-20.5 FIG S4 Proportion of women authors (*x* axis) on submitted manuscripts (*y* axis, log_10_ scale) according to the number of coauthors (individual plot) and the gender of the corresponding author (orange/blue). Single-author papers were eliminated, and the manuscripts were grouped according to the number of coauthors: groups of 5 authors up to 20 authors, groups of 10 authors up to 40 authors, and a single group of manuscripts with 40+ authors. The manuscripts were then split according to the proportion of coauthors that were inferred to be women: 0, up to 24%, 25 to 50%, 51 to 75%, and more than 75%. The manuscripts were then further split according to the inferred gender of the corresponding author. Regardless of the number of coauthors, women corresponding authors (orange) submitted more manuscripts with a majority (>50%) of women coauthors than men corresponding authors. Men corresponding authors submitted more manuscripts with less than 50% women coauthors than women corresponding authors did, and the trend of this gap increased with the number of coauthors. Download FIG S4, TIF file, 0.2 MB. Copyright © 2020 Hagan et al. 2020 Hagan et al. This content is distributed under the terms of the Creative Commons Attribution 4.0 International license.

We hypothesized that we would be able to predict the inferred gender of the corresponding author using a logistic regression model trained on the following variables: whether the corresponding author’s institution was in the United States, the total number of authors, the proportion of authors that were women, whether the paper was published, the gender of senior editors and editors, the number of revisions, and whether the manuscript was editorially rejected at any point. We measured the model’s performance using the area under the receiver operating characteristic curve (AUROC). The AUROC value is a predictive performance metric that ranges from 0.0, where the model’s predictions are completely wrong, to 1.0, where the model distinguishes perfectly between outcomes. A value of 0.5 indicates that the model did not perform better than a random assignment. The median AUROC value of our model to predict the corresponding author’s inferred gender was 0.7 (Fig. S5A, column A). The variable with the largest absolute weight (i.e., the most predictive value), in our model was the proportion of women authors (Fig. S5C). These results indicate that manuscript submission data were capable of predicting the inferred gender of the corresponding author but that the prediction was primarily driven by the percentage of authors that were inferred to be women.

10.1128/mBio.01680-20.6 FIG S5 Box plots of linear regression results from 25 data splits. (A) AUC values. Each column represents a different prediction model: A, corresponding author’s gender; B, author gender on editorial decisions; C, institution on editorial decisions. (B and C) Effect of variables on the logistic regression model represented as the absolute values of the variable weight. (B) Author gender from editorial decisions. (C) Corresponding author’s gender. Download FIG S5, TIF file, 0.5 MB. Copyright © 2020 Hagan et al. 2020 Hagan et al. This content is distributed under the terms of the Creative Commons Attribution 4.0 International license.

As described above, first authors were slightly more likely to be women (30.7%W versus 29.1%M), but corresponding authors were significantly more likely to be men (23.44%W versus 41.59%M). A concern is that if authors are not retained to transition from junior to senior status, they will be left out of the gatekeeping roles. Since authorship conventions indicate that the last and corresponding authors are typically senior authors, we combined both first and middle authors into the “junior” author role and used the unique identifiers assigned to each account to track individuals through the possible roles at ASM journals. There were 75,451 women who participated as junior authors (first/middle) at ASM journals. Of those junior authors who were women, 8.2% also participated as senior authors (last/corresponding), 8.9% were potential reviewers, and 5.4% participated as reviewers. 0.2% of women junior authors became editors at ASM journals over the 6-year period studied. For men, there were a total of 83,727 junior authors, where 13.6% also participated as senior authors, 16.7% were potential reviewers, and 11.1% actually reviewed. 0.7% of men junior authors became editors at the ASM journals. Overall, women who participated at ASM journals as junior authors were half as likely to move to senior author or reviewer roles and 30% as likely to be an editor than men at ASM journals.

### Manuscripts submitted by women have more negative outcomes than those submitted by men.

To further investigate the difference in percentages of published and submitted proportions for men and women authors (Fig. 4C and D and Fig. S3), we compared the rejection rates of men and women at each author stage (first, middle, corresponding, and last). To more easily visualize and understand the differences in outcomes according to author gender, we calculated the outcome rate for each gender and then subtracted the rate for women from the rate for men to generate the percentage point difference. To correct for the disparity in participation by women compared to men, all percentage point comparisons were made relative to the gender and population in question. Where a decision favored men (is biased against women), the value of the difference in percentage points was on the right (blue), while values on the left (orange) indicate the number of percentage points that women outperformed men in the given metric. For the following analyses, only manuscripts authored by an individual inferred to be a man or woman were included. Finally, these analyses were conducted on all available manuscripts, not a statistical sampling. As a result, statistical tests were required only for correlative analyses.

**FIG 5:**
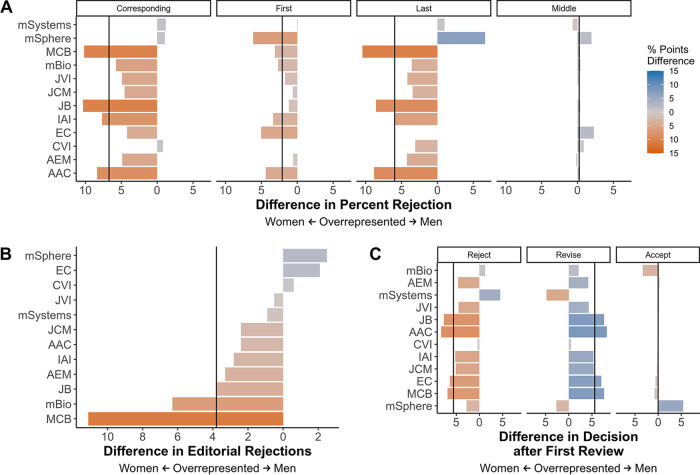
Difference in manuscript outcomes by author gender. The difference in the percentage of manuscript outcomes was calculated by subtracting the percentage of women who received the outcome from the percentage of men who received the outcome. Values on the left (orange) are percentage point differences indicating that women received the outcome more often, 0 (or no bar) indicates equal rates of the outcome, and values on the right (blue) indicate the number of percentage points that men received the outcome more frequently. Vertical lines indicate the difference value for all journals combined. (A) Difference in percent rejections by author gender and type (e.g., corresponding, first, last, middle) at any stage across all journals. (B) Difference in percent editorial rejection rates for corresponding authors at each journal. (C) Difference in percentage points between each decision type for corresponding authors following the first peer review.

Middle authors were rejected at equivalent rates for men and women (a 0.23 percentage point difference across all journals). However, manuscripts with senior women authors were rejected more frequently than those authored by men with 6.7 and 6.0 percentage point differences for corresponding and last authors, respectively (Fig. 5A, vertical lines). The overall trend of increased rejection for women was most pronounced at MCB, JB, IAI, and AAC. The greatest differences were observed when comparing the outcome of corresponding authors by gender, so we used this subpopulation to further examine the difference in manuscript acceptance and rejection rates between men and women.

We next compared the rejection rates for men and women corresponding authors after two review points, initial editor review and the first round of peer review. Manuscripts authored by women were editorially rejected by as much as 12 percentage points more often than those authored by men (Fig. 5B). The difference at all of the ASM journals combined favored men by 3.8 percentage points (vertical line). MCB and *mBio* had the most extreme percentage point differences. Manuscripts authored by men and women were equally likely to be accepted after the first round of review (Fig. 5C, right column). However, women-authored papers were rejected (left column) more often than men-authored papers by 5.6 percentage points. Meanwhile, men-authored papers were given revision (Fig. 5C, center column) decisions 5.6 percentage points more frequently than women (Fig. 5C, vertical lines). JB, AAC, and MCB had the most extreme differences for rejection and revision decisions. Percentage point differences were not correlated with journal prestige as measured by 2018 impact factors (*R*^2^ = −0.022, *P* = 0.787).

In addition to manuscript decisions, other disparate outcomes may occur during the peer review process (29). To determine whether accepted women-authored manuscripts spent more time between being submitted and being ready for publication, we compared the number of revisions, days spent in the ASM peer review system, and the number of days between submission and being ready for publication to those authored by men. Manuscripts authored by women took slightly longer to complete than those by men at all journals, an additional 1 to 9 days on average from submission to ready for publication (Fig. S6A). This was despite spending similar amounts of time in the ASM journal peer review system (from 1 day less to 4 days more than men) (Fig. S6B) and having the same median number of revisions prior to acceptance (median = 2; interquartile range [IQR] = 0).

10.1128/mBio.01680-20.7 FIG S6 Comparison of time to final accepted decision and time spent in the peer review system by journal and gender. The number of days between when a manuscript is initially submitted and officially accepted (A) or that a manuscript spends in the ASM peer review system (i.e., the sum of days from author submission to editor decision for each submitted version) (B). Download FIG S6, TIF file, 0.4 MB. Copyright © 2020 Hagan et al. 2020 Hagan et al. This content is distributed under the terms of the Creative Commons Attribution 4.0 International license.

**FIG 6:**
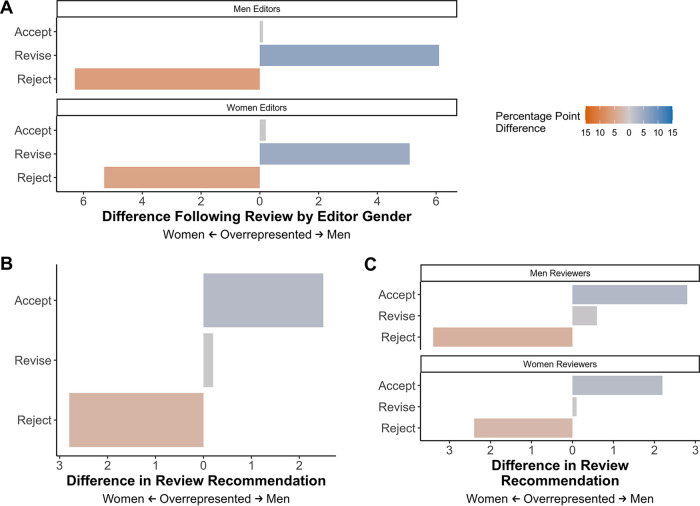
Difference in decisions or recommendations according to the gatekeeper gender. The difference in the percentage of manuscript outcomes was calculated by subtracting the percentage of women who received the outcome from the percentage of men who received the outcome. Values on the left (orange) are percentage point differences indicating that women received the outcome more often, 0 (or no bar) indicates equal rates of the outcome, and values on the right (blue) indicate the number of percentage points that men received the outcome more frequently. (A) Effect of editor gender on the difference in decisions following review. (B and C) Difference in percentage points for review recommendations (B) and how that is affected by reviewer gender (C). Panels A to C show all journals combined.

To understand how a gatekeeper’s (editor/reviewer) gender interacted with decision types (e.g., Fig. 5C), we grouped editor decisions and reviewer suggestions according to the gatekeeper’s inferred gender (unknowns excluded). Both men and women editors rejected proportionally more women-authored papers; however, the percentage point difference in decisions were slightly larger for men-edited manuscripts (Fig. 6A). Reviewers were more likely to suggest rejection for women-authored manuscripts compared to men-authored manuscripts, and a minimal difference in revise recommendations was observed (Fig. 6B). Both men and women reviewers recommended rejection more often for women-authored manuscripts, although men recommended acceptance and revision more frequently for men-authored manuscripts than women did (Fig. 6C).

To evaluate whether inferred gender played a role in manuscript editorial decisions, we trained a logistic regression model to predict whether a manuscript was reviewed (i.e., editorially rejected or not). We used the inferred genders of the senior editor, editor, and corresponding author, as well as the proportion of authors that were women as variables to train the model (Fig. S5B). The median AUROC value was 0.61 (Fig. S5A, column B), which indicated that editorial decisions were not random; however, the relatively low AUROC value indicated that there are factors not included in our model that influence editorial decisions.

### Multiple factors contribute to the overperformance of men.

The association between inferred gender and manuscript decision could be attributed to implicit gender bias by journal gatekeepers; however, there are other types of bias that may contribute to, or obscure, gender bias; for instance, a recent evaluation of peer review outcomes at *eLife* found evidence of preference for research submitted by authors from a gatekeeper’s own country or region (20). Other studies have documented prestige bias, where men are overrepresented in more prestigious (i.e., more respected and selective) programs (30). It is therefore possible that what seems to be gender bias could be geographic or prestige bias interacting with the increased proportion of women submitting from outside the United States or from lower prestige institutions (e.g., the highest rate of submissions from women were at low research institutions, 37%; Fig. 4A).

**FIG 7:**
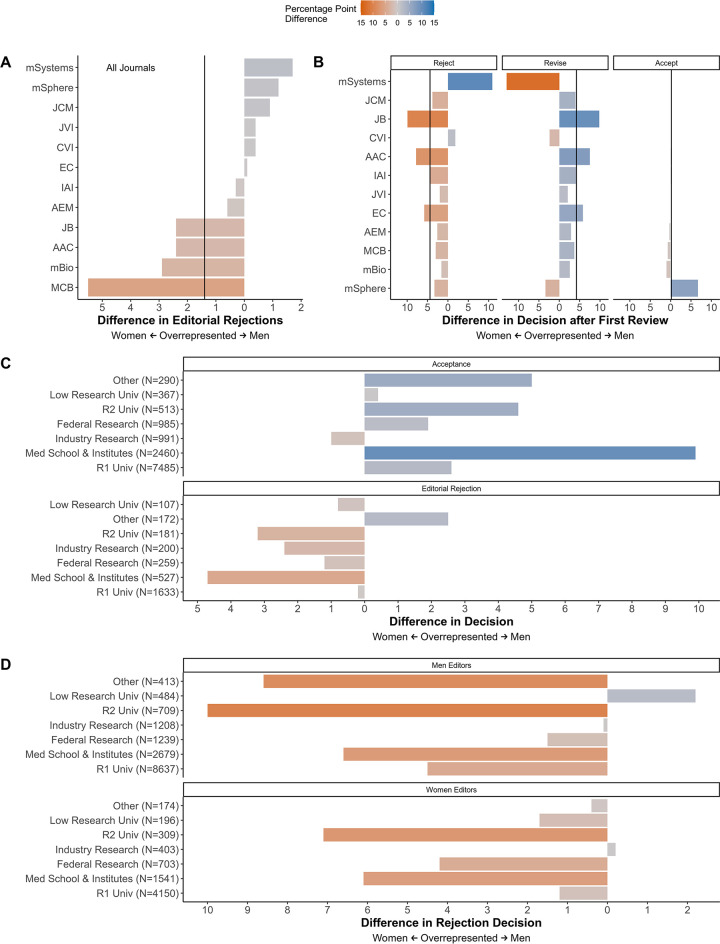
Impact of origin and U.S. institution type on manuscript decisions by gender. The difference in the percentage of manuscript outcomes was calculated by subtracting the percentage of women who received the outcome from the percentage of men who received the outcome. Values on the left (orange) are percentage point differences indicating that women received the outcome more often, 0 (or no bar) indicates equal rates of the outcome, and values on the right (blue) indicate the number of percentage points that men received the outcome more frequently. Vertical lines indicate the difference value for all of the ASM journals combined. (A and B) Difference in percentage points for editorial rejections (A) and following first review of manuscripts (B) submitted by U.S.-based corresponding authors. (C and D) Difference in percentage points for acceptance and editorial rejections according to institution types (C) and rejection decisions by editor gender and institution type (D).

To quantify how these factors affected manuscript decisions, we next looked at the outcome of manuscripts submitted only by corresponding authors at U.S. institutions, because these institutions represented the majority of manuscripts and could be classified by using the Carnegie Classification of Institutions of higher education (27). We used the same strategy as described above. When only considering U.S.-based authors, the bias in editorial rejections against papers submitted by women decreased from 3.8 to 1.4 percentage points (Fig. 7A). The trend of percentage point difference in decisions after review for U.S.-based authors mirrored those seen for all corresponding authors at the journal level (Fig. 7B). The overrepresentation of women in rejection decisions decreased from 5.6 to 4.4 percentage points, and the overrepresentation of men in revise only decisions decreased from 5.6 to 4.2, moving manuscript outcomes toward parity (Fig. 7B). The difference in the rate of accept decisions changed from favoring men 1.4 to favoring women 0.2 percentage points after restricting the analysis to U.S.-based authors, indicating near equal acceptance for corresponding authors of both genders. These results suggest that the country of origin (i.e., U.S. versus not U.S.) accounted for some of the differences in outcomes by inferred gender, particularly for editorial rejections.

To address institution-based prestige bias, we split the U.S.-based corresponding authors according to the type of institution they were affiliated with (based on the Carnegie Classification) and reevaluated the differences for men and women (27). Editorial rejections occurred most often for women from medical schools or institutes, followed by those from R2 institutions: 32% and 28% of manuscripts from each institution were submitted by women, respectively (Fig. 7C and Fig. S7A). This percentage point difference in the editorial rejections of corresponding authors from medical schools or institutes was spread across most of the ASM journals, while the editorial rejection of papers submitted from women at R2 institutions was driven primarily by submissions to JCM (Fig. S7A). Evaluating the percentage point difference in acceptance rates by institution and inferred gender mirrored that of editorial rejections for some journals, where submissions from men received better outcomes than submissions from women (Fig. 7C and D and Fig. S7B and C). For instance, manuscripts submitted by men from medical schools or institutes were accepted up to 10 percentage points more often than those submitted by women (Fig. 7C).

10.1128/mBio.01680-20.8 FIG S7 Difference in editorial rejection (A) and acceptance (B) rates by journal and institution type. (C) Difference in review recommendations by reviewer gender and author institution type. (D) Median importance (black dot) of features affecting editorial rejections, and their range. The color of smaller dots (*n* = 25) indicates the direction of the impact. Download FIG S7, TIF file, 1.0 MB. Copyright © 2020 Hagan et al. 2020 Hagan et al. This content is distributed under the terms of the Creative Commons Attribution 4.0 International license.

To evaluate whether these factors affect manuscript decisions, we trained a logistic regression model to predict whether a manuscript was editorially rejected using the following variables: origin (U.S. versus non-U.S.), institution (U.S. institution type), number of authors, proportion of authors that were women, and the inferred genders of both gatekeepers and corresponding authors. The model had a median AUROC value of 0.67 (Fig. S5A, column C), which indicated a nonrandom interaction between these factors and editorial decisions. Manuscripts from authors at “other” U.S. institutions, men EICs, men that were corresponding authors from “other” U.S. institutions, and women from medical schools and institutes were all more associated with editorial rejections (Fig. S7D). Conversely, manuscripts from R1 institutions, authors from the United States, EICs that were women, and the number of authors were all more likely to be associated with review (Fig. S7D). These results confirm that the country of origin and class of institution impact decisions in a nonrandom manner, though not as much as gender.

A final factor we considered was whether the type of research pursued by men as opposed to women may impact manuscript outcomes. Black women philosophers and physicists have described the devaluation of nontraditional subdisciplines in their fields (31–33). This concept originally described bias against Black women—the intersection of two historically marginalized identities. However, the idea that researchers in an established core field might be skeptical of less established, or nontraditional, subfield research likely applies elsewhere. The disparate outcomes of subfields in a gendered context has recently been observed in the biomedical sciences, where NIH proposals focusing on women’s reproductive health were the least likely to be funded (34). To explore this phenomenon in ASM journals, we looked at the editorial rejection rates of manuscripts (regardless of origin or institution) for each research category at the five largest ASM journals: AAC, AEM, IAI, JVI, and JCM. Together, these journals account for 47% of the manuscripts analyzed in this study and comprise 55 categories.

The number of submissions in each category ranged from 1 (“FDA Approval” at AAC) to 2,952 (“Bacteriology” at JCM), while the acceptance rates varied from 29.4% (“Chemistry: Biosynthesis” at AAC) to 71.3% (“Structure and Assembly” at JVI) (Table 1). We argued that the number of submissions to each category could help indicate core versus periphery subfields, (i.e., core subfields would have more submissions than periphery subfields) and based on the literature to date, we expected that periphery subfields might have a higher participation of women (31–33). Women submitted on average 35.3% of the manuscripts to each category, ranging from 20% to 86% (Table 1). There was not a correlation between the proportion of women authors and the number of submissions (*R*^2^ = −0.0177, *P* = 0.779) to each category. Nor was there a correlation between the proportion of women authors and the category acceptance rate (*R*^2^ = 0.041, *P* = 0.078). These data suggest that there was not a relationship between the participation of women and either the number of submissions or the acceptance rate of categories in our data set.

**TABLE 1:** Analysis of subdiscipline participation by women corresponding authors at five ASM journals

Journal	Category	*n*	% accepted^*a*^	% women editors^*a*^	% women authors^*a*^
AAC	Analytical Procedures	135	43.0	14	29
AAC	Antiviral Agents	836	56.5	6	33
AAC	Biologic Response Modifiers	44	40.9	12	43
AAC	Chemistry: Biosynthesis	109	29.4	10	32
AAC	Clinical Therapeutics	1,060	48.9	13	31
AAC	Epidemiology and Surveillance	765	52.3	14	40
AAC	Experimental Therapeutics	1,329	57.4	13	28
AAC	FDA Approvals	1	NA	NA	NA
AAC	Mechanisms of Action: Physiological Effects	597	51.8	14	30
AAC	Mechanisms of Resistance	1,783	60.0	14	36
AAC	Pharmacology	878	66.6	13	29
AAC	Susceptibility	1,051	46.8	12	39
AEM	Biodegradation	302	38.4	35	26
AEM	Biotechnology	802	37.9	30	27
AEM	Environmental Microbiology	2,395	30.3	35	42
AEM	Enzymology and Protein Engineering	340	46.5	28	24
AEM	Evolutionary and Genomic Microbiology	279	48.4	32	30
AEM	Food Microbiology	1,216	38.2	33	39
AEM	Genetics and Molecular Biology	587	51.8	32	36
AEM	Geomicrobiology	151	44.4	34	37
AEM	Invertebrate Microbiology	317	45.7	29	37
AEM	Methods	529	39.7	30	29
AEM	Microbial Ecology	1,121	35.8	29	37
AEM	Mycology	73	47.9	33	44
AEM	Physiology	356	50.3	32	31
AEM	Plant Microbiology	346	36.4	29	39
AEM	Public and Environmental Health Microbiology	893	34.0	32	45
IAI	Bacterial Infections	716	58.4	35	36
IAI	Cellular Microbiology: Pathogen-Host Cell Molecular Interactions	685	55.2	35	37
IAI	Fungal and Parasitic Infections	353	59.5	33	33
IAI	Host Response and Inflammation	763	50.2	35	40
IAI	Host-Associated Microbial Communities	7	57.1	43	86
IAI	Microbial Immunity and Vaccines	342	56.4	35	32
IAI	Molecular Genomics	33	60.6	37	33
IAI	Molecular Pathogenesis	617	68.4	35	31
JCM	Bacteriology	2,952	33.2	27	41
JCM	Chlamydiology and Rickettsiology	80	32.5	25	41
JCM	Clinical Veterinary Microbiology	364	32.7	29	40
JCM	Epidemiology	854	29.7	30	45
JCM	Fast-Track Communications	5	40.0	33	40
JCM	Immunoassays	139	36.0	31	41
JCM	Mycobacteriology and Aerobic Actinomycetes	510	42.9	32	41
JCM	Mycology	587	37.3	19	39
JCM	Parasitology	337	33.2	27	34
JCM	Virology	1,140	37.5	29	41
JVI	Cellular Response to Infection	604	51.2	36	32
JVI	Gene Delivery	98	41.8	32	20
JVI	Genetic Diversity and Evolution	883	51.1	39	27
JVI	Genome Replication and Regulation of Viral Gene Expression	813	64.6	39	23
JVI	Pathogenesis and Immunity	1,622	60.4	35	33
JVI	Prions	92	69.6	56	22
JVI	Structure and Assembly	725	71.3	39	29
JVI	Transformation and Oncogenesis	154	59.1	39	36
JVI	Vaccines and Antiviral Agents	1,149	59.2	36	28
JVI	Virus-Cell Interactions	2,414	63.6	40	30

a NA, not available.

We next looked at the percentage point differences in performance for men and women in each category at two decision points: editorial rejection and rejection after the first review. Each journal focuses on a different facet of microbiology or immunology, making the results difficult to compare directly. However, the pattern of increased rejection rates for women was maintained across most categories with some displaying major differences in gendered performance (Fig. S8). For instance, the “Biologic Response Modifier” (e.g., immunotherapy) subcategory at AAC had extreme differences for both editorial rejections and rejections after review where men were favored by 30 and 40 percentage points, respectively. While that category had a relatively low number of submissions (*n* = 44), 43% were from women (Fig. S8A). “Mycology” was a category at two journals, AEM and JCM. At both journals, men received favorable outcomes relative to women in this category. At AEM, there were 73 “Mycology” submissions, 44% from women authors with an almost 20 percentage point difference favoring the editorial rejection outcomes of men corresponding authors. Men authors were slightly less favored in rejections after review at a 10 percentage point difference (Fig. S8B). JCM had 587 “Mycology” submissions with a submission rate of 39% from women authors (Fig. S8D). Differences between JCM “Mycology” outcomes also favored men authors by almost 10 and 12 percentage points for editorial rejections and rejections after review, respectively.

10.1128/mBio.01680-20.9 FIG S8 Difference in editorial rejections and rejections after review by corresponding author gender and manuscript category at AAC (A), AEM (B), IAI (C), JCM (D), and JVI (E). The number of manuscripts submitted is shown by N in parentheses. Download FIG S8, TIF file, 1.1 MB. Copyright © 2020 Hagan et al. 2020 Hagan et al. This content is distributed under the terms of the Creative Commons Attribution 4.0 International license.

Because of these extreme percentage point differences in categories with high women authorship, we next asked whether the number of women participating in a particular category was related to manuscript outcomes. There was no correlation between the difference in editorial rejection by category and the percentage of women that were either authors (*R*^2^ = −0.003, *P* = 0.363) or editors (*R*^2^ = −0.018, *P* = 0.765). The percentage of women authors and percentage of women editors in journal categories did not correlate either (*R*^2^ = −0.007, *P* = 0.682), which is likely related to the underrepresentation of women editors in categories dominated by women authors (e.g., “Epidemiology”). These data suggest the possibility of persistent negative outcomes against women in particular fields (e.g., “Mycology”), though it does not seem to relate to either the number of submissions or participation of women in those subfields.

## DISCUSSION

We described the representation of inferred men and women participating in the submission and peer review process at ASM journals between January 2012 and August 2018 and compared editorial outcomes according to the authors’ inferred gender. Women were consistently underrepresented (30% or less in all levels of the peer review process) excluding first authors, where women represented about 50% of authors where we could infer a gender (Fig. 2 and 4). Women and men editors had proportionate workloads across all of the ASM journals combined, but those workloads were disproportionate at the journal level and the overburdened gender varied by journal (Fig. 2 and 3). Additionally, manuscripts submitted by women corresponding authors received more negative outcomes (e.g., editorial rejections) than those submitted by men (Fig. 5 and 6). These negative outcomes were somewhat mediated by whether the corresponding author was based in the United States, the type of institution for U.S.-based authors, and the research category (Fig. 7 and Fig. S8). However, the trend for women corresponding authors to receive more negative outcomes held across all analyses, indicating a pattern of gender-influenced editorial decisions regardless of journal prestige (as determined by impact factor). Together, these data indicate a persistent penalty for senior women microbiologists who participate at ASM journals.

How to define representation and determine what the leadership should look like are recurring questions in STEM (science, technology, engineering, and mathematics). Ideally, the representation for men and women corresponding authors, reviewers, and editors would reflect the number of Ph.D.’s awarded (about 50% each, when considered on a binary spectrum). We argue that the goal should depend on the workload and visibility of the position. Since high visibility positions (e.g., editor, EIC) are filled by a smaller number of individuals that are responsible for recruiting more individuals into leadership, filling these positions should be done aspirationally (i.e., 50% should be women if the goal were an aspirational leadership). This allows greater visibility for women as experts, expansion of the potential reviewer network, and recruitment into those positions (35–37). Conversely, lower visibility positions (e.g., reviewers) require effort from a greater number of individuals and should thus be representational of the field to avoid overburdening the minority population (i.e., since 23.5% of corresponding authors at the ASM journals are women, then 20 to 25% of reviewers should be women). Balancing the workload is particularly important given the literature indicating that women faculty have higher institutional service loads than their counterparts who are men (38).

Our data also revealed some disturbing patterns in gendered authorship that have implications for the retention of women microbiologists. Previous research suggests that women who collaborate with other women receive less credit for these publications than when they collaborate with men (39) and that women are more likely to yield corresponding authorship to colleagues that are men (21). In our linear regression models, the number of authors on a manuscript was the largest contributor to avoiding editorial rejections, suggesting that highly collaborative research is preferred by editors (see Fig. S7D in the supplemental material). This observation was supported by the positive correlation between citations and author count (40). Thus, it concerns us that when the number of authors exceeded 30 on a manuscript (*n* = 59), the proportion of individuals inferred to be women was always below 51%, despite equivalent numbers of trainees in the biological sciences (Fig. S4). While women corresponding authors submitted fewer manuscripts, more of their papers (both numerically and proportionally) had a majority of coauthors inferred to be women compared to those submitted by men corresponding authors. These data support previous findings that women are more likely to collaborate with other women (23, 41–43). Additionally, the proportion of women authors was the greatest predictor of corresponding author gender. This gender-based segregation of collaborations at ASM journals likely has had consequences in pay and promotion for women microbiologists and could be a factor in the decreased retention of senior women. We predict that the low retention is aggravated by the underrepresentation of women as corresponding authors, which also has negative consequences for both their careers and microbiology. Since senior authorships impact status, visibility, and salary, the underrepresentation of women as senior authors and reviewers likely hampers their career progression and desire to progress (18, 44). The retention of women (and other marginalized groups) is important to the progress of microbiology since less diversity in science limits the diversity of perspectives and approaches, thus stunting the search for knowledge.

Even if a gatekeeper does not know the corresponding author or their gender, there remain ample avenues for implicit bias during peer review. The stricter standard of competency has led women to adopt different writing styles from men, resulting in manuscripts with increased explanations, detail, and readability than those authored by men (29, 45). Additionally, women are often at a disadvantage for the resources required for highly competitive fields due to cumulative penalties (9–11). As a result, corresponding authors that are women may be spending their resources in research fields where competition impacts are mitigated and/or on topics that are historically understudied; thus, these are cues to gender and perceived competency (31–33). Alternatively, nontraditional research may be seen as less impactful, leading to poorer peer review outcomes (34). These possibilities are reflected in our data, since while the number of revisions before publication is identical for both men and women, manuscripts authored by women have increased rejection rates and time spent on revision. This suggests that manuscripts submitted by women receive more involved critiques (i.e., work) from reviewers and/or their competency to complete revisions within the prescribed 30 days is doubted compared to men. Women may also feel that they need to do more to meet reviewer expectations, thus leading to longer periods between a decision and resubmission. Finally, our data show a penalty for women researching mycology (Fig. S8). Despite being among the most deadly infectious diseases in 2016 (along with tuberculosis and diarrheal diseases), mycology is an underserved, and underfunded, field in microbiology that has historically been considered unimportant (46–49). Microbiology would benefit from a more nuanced evaluation of subfields to better understand how they interact with gender and peer review outcomes.

A limitation to our methodology is the use of an algorithm to infer gender from first names. While we report a high accuracy (0.97 to 0.99) where gender was inferred, this method left us with a category of unknown gendered individuals. Additionally, the gender of an individual may be interpreted differently according to the reader (e.g., Kim is predominately a woman’s name in the United States, but likely a man’s name in other cultures). The increase in unknown gendered authors corresponds to an increase in submissions to ASM journals from Asian countries, particularly China. Anecdotally, most editorial rejections are poor quality papers from Asia, and our method had low performance on nongendered languages from this region (see Text S1 and Fig. S1 in the supplemental material), thus excluding many Asia-submitted manuscripts and increasing our confidence that the trends observed were gender based. For corresponding authors, manuscript submissions are the end product of several other prior decisions such as a mentor’s implicit bias(es), postdoctoral fellowships, faculty applications, start-up funding negotiations, and grant proposals. These prior factors, which cannot be accounted for in our analysis, along with the small effect size observed in some analyses, limit quantifying the role of gatekeeper decisions in the disparate gender-influenced outcomes. However, the consistency of decisions to benefit men corresponding authors over women across all of the journals included in this study, in addition to accumulated literature thus far, confirms that this descriptive study is highly relevant for the ASM as a society. Our findings offer opportunities to address gendered representation in microbiology and systemic barriers in peer review at our journals.

All parties have an opportunity and obligation to advance marginalized groups in science (50, 51). We suggest that journals develop a visible mission, vision, or other statement that commits to equity, justice, and inclusion and includes a nondiscrimination clause regarding decisions made by editors and editors-in-chief. This nondiscrimination clause should be backed by a specific protocol for the reporting of, and response to, instances of discrimination and harassment. Second, society journals should begin collecting additional data from authors and gatekeepers such as race, ethnicity, gender identity, and disabilities. These data should not be available to journal gatekeepers but instead should be kept in a disaggregated manner that allows for public presentation, tracking the success of inclusive measures, and to maintain accountability. Third, society journals can implement mechanisms to explicitly provide support for women and other marginalized groups, reward inclusive behavior by gatekeepers, nominate more women to leadership positions, and recruit manuscripts from subfields that are more likely to attract women and other marginalized groups (34). We can all help advance women (and other marginalized groups) within the peer review system by changing how we select experts in our field. For instance, authors can suggest more women as reviewers using “Diversify” resources (52), while reviewers can agree to review for women editors more often. Editors can rely more on manuscript reference lists and database searches than personal knowledge to recruit reviewers (53), and journals can improve the interactivity and functionality of the reviewer selection software. Given the propensity for journals to recruit editors and EICs from within their already skewed reviewer pools, opening searches to include more scientists in their reviewer pool and/or editors from outside the journal while enacting more transparent processes could be a major component of improving representation. Growing evidence suggests that representation problems in STEM are due to retention rather than recruitment. We need to align journal practices to foster the retention of women and other marginalized groups.

Most approaches to disparate outcomes focus on choices made by individuals, such as double-blinded reviews and implicit bias training. These cannot fully remedy the effects of implicit bias and may even worsen outcomes (54, 55). Since disparate outcomes (by gender, geography, prestige, or otherwise) are primarily the result of accumulated disadvantages and actions resulting from implicit biases and systemic “-isms,” a structural, system-wide approach is required (56–58). Broadly, peer review is a nebulous process with expectations and outcomes that vary considerably, even within a single journal. Academic writing courses suffer similar issues and have sought to remedy them with rubrics. When implemented correctly, rubrics can reduce implicit bias during evaluation and enhance the evaluation process for both the evaluator and the evaluatee (59–62). We argue that rubrics could be implemented in the peer review process to focus reviewer comments, clarify editorial decisions, and improve the author experience. Such rubrics should increase the emphasis on solid research, as opposed to novel or “impactful” research, the latter of which is a highly subjective measure (63, 64). This might also change the overall negative attitude toward replicative research and negative results, thus bolstering the field through reproducibility. We also argue that reconsidering journal scope and the membership of honorary editorial boards might help address structural penalties resulting from implicit bias against women (and other marginalized groups) in peer review. Expanding journal scope and adding more handling editors would improve the breadth of research published, thus providing a home for more nontraditional and underserved research fields (the case at *mSphere* with an increased pool of editors). Implementing these steps to decrease implicit bias and structural penalties—review rubrics, increased focus on solid research, expansion of journal scopes and editorial boards—will also standardize competency principles for researchers at ASM journals and improve microbiology as a whole.

Although the level of bias at many of the ASM journals is small, it is present. Peer review at ASM journals is not immune to the accumulated disadvantages against women in microbiology. However, the adaptation of women and other marginalized groups to implicit bias (e.g., area of research and communication styles) make it impossible to address at the individual level. Instead, we must commit to changing the fundamental structure and goals of peer review to minimize the impact of such bias. We encourage ASM journals, as well as other societies, to institute more fair and transparent procedures and approaches of peer review. The self-correcting nature of science is a badge that scientists wear proudly, but no single report or action can correct the inertia of a centuries-old institution. Instead, it requires the long-standing and steady actions of many. Our findings reflect many similar reports, and we suggest concrete actions to correct the inertia of peer review at all levels. The next steps are commitment and implementation.

## DATA AND METHODS

### Data.

All manuscripts handled by ASM journals (e.g., *mBio*, *Journal of Virology*) that received an editorial decision between 1 January 2012 and 31 August 2018 were supplied as XML files by the ASM’s publishing platform, eJP. Data were extracted from the XML documents provided, manipulated, and visualized using R statistical software (version 3.4.4) and relevant packages. Variables of interest included the manuscript number assigned to each submission, manuscript type (e.g., full-length research, erratum, editorial), category (e.g., microbial ecology), related (i.e., previously submitted) manuscripts, number of versions submitted, dates (e.g., submission, decision), author data (e.g., first, last, and corresponding authorship, total number of authors), reviewer data (e.g., recommendation, editor decision), and personal data (names, institutions, country) of the editors, authors, and reviewers. Since reviews and commentaries are often commissioned, only original, research-based manuscripts were included in this analysis, e.g., long- and short-form research articles, New-Data Letters, Observations, Opinion/Hypothesis articles, and Fast-Track Communications. To help protect the confidentiality of peer review, names were removed from all records, and identifying data (e.g., manuscript numbers, days of date) were replaced with randomized values.

### Institution classification.

To identify the communities represented, we used the Carnegie classifications to group U.S.-based academic institutions into R1 research (very high research activity), R2 research (high research activity), 4-year medical schools, or low research (i.e., not R1, R2, or medical school) (27). Research institutes (e.g., Mayo Clinic, Cold Springs Harbor), industry (e.g., pharmaceutical), and federal (e.g., NIH, CDC) research groups were identified using the Internet. Four-year medical schools and research institutions were grouped together since these typically share research prestige and have considerable resources to support research. Industry and federal research were their own groups. The “Other” category represents uncategorized U.S. institutions. Non-U.S. institutions were their own category.

### Gender inference.

The genderize.io API was used to infer an individual’s gender based on their given name and country where possible. The genderize.io platform uses data gathered from social media to infer gender based on given names with the option to include an associated language or country to enhance the probability of successful inference. Since all manuscripts were submitted in English, which precludes language association for names with special characters, names were standardized to ASCII coding (e.g., “José” to “Jose”). We next matched each individual’s country against the list of 242 country names accepted by genderize.io. Using the GenderGuesser package for R, all unique given names associated with an accepted country were submitted to the genderize.io API and any names returned without an inferred value of either male or female were resubmitted without an associated country. The data returned include the name, inferred gender (as “male,” “female,” or “unknown”), the probability of correct gender inference (ranging from 0.5 to 1.0), and the number of instances the name and gender were associated together (1 or greater). The inferred genders of all given names (with and without an associated country) whose probabilities were greater or equal to a modified probability (pmod) of 0.85 were used to infer genders (man/woman) of the individuals in our data set (Text S1). The presenting gender (man/woman) of editors and senior editors in our data set was inferred by hand using Google where possible, and the algorithm was validated using both editor and published data (Text S1) (5).

### Manuscript outcome analysis.

To better visualize and understand the differences in outcomes according to author gender, we calculated the difference in percentage points between the proportion of that outcome for men and women. To correct for the disparity in the participation of women relative to men at ASM journals, all percentage point comparisons were made relative to the gender and population in question. For instance, the percentage point difference in acceptance rates was the acceptance rate for men minus the acceptance rate for women. A positive value indicated that men received the outcome more often than women, whereas a negative value indicated that women outperformed men in the given metric. Data are presented as divergent absolute values.

### Logistic regression models.

For the L2-regularized logistic regression models, we established modeling pipelines for a binary prediction task (65). First, we randomly split the data into training and test sets so that the training set consisted of 80% of the full data set while the test set was composed of the remaining 20% of the data. To maintain the distribution of the two model outcomes found with the full data set, we performed stratified splits. The training data were used to build the models, and the test set was used for evaluating predictive performance. To build the models, we performed an internal fivefold cross-validation where we tuned the cost hyperparameter, which determines the regularization strength where smaller values specify stronger regularization. This internal cross-validation was repeated 100 times. Then, we trained the full training data set with the selected hyperparameter values and applied the model to the held-out data to evaluate the testing predictive performance of each model. The data split, hyperparameter selection, training, and testing steps were repeated 25 times to obtain a reliable and robust reading of model performance. Models were trained using the machine learning wrapper caret package (v.6.0.81) in R (v.3.5.0).

### Code and data availability.

Data and code for all analysis steps, logistic regression pipeline, and an Rmarkdown version of this article, are available at https://github.com/SchlossLab/Hagan_Gender_mBio_2020/.
