# Erratum for Hagan et al., “Women Are Underrepresented and Receive Differential Outcomes at ASM Journals: a Six-Year Retrospective Analysis”

**DOI:** 10.1128/mBio.00355-21

**Published:** 2021-03-23

**Authors:** Ada K. Hagan, Begüm D. Topçuoğlu, Mia E. Gregory, Hazel A. Barton, Patrick D. Schloss

**Affiliations:** a Department of Microbiology and Immunology, University of Michigan, Ann Arbor, Michigan, USA; b Department of Biology, University of Akron, Akron, Ohio, USA

## ERRATUM

Volume 11, no. 6, e01680-20, 2020, https://doi.org/10.1128/mBio.01680-20. The first paragraph of the Acknowledgments (PDF page 19) section should have read as follows:

We thank Nicole Broderick and Arturo Casadevall for providing their data set for genderize validation and Joel F. Plotkin (eJournalPress) for his assistance in providing us with the XML files that were analyzed in this study. We are grateful to Arianna Miles-Jay and Joshua M. A. Stough for their comments.

