# Supplementary material for: Women Are Underrepresented and Receive Differential Outcomes at ASM Journals: a Six-Year Retrospective Analysis
Source: mBio. 2020 Dec 1;11(6):e01680-20. doi: 10.1128/mBio.01680-20 (PMC7733940; doi:10.1128/mBio.01680-20)
Supplement: TEXT S1 [file mBio.01680-20-s0001.pdf]

# Supplementary Text

**Validation of gender inference.** We first validated the genderize.io algorithm using a set of 3265 names whose gender had been hand-coded based on appearance (1). The names were supplied to the genderize algorithm both with and without the accompanying country data. The genderize algorithm returned gender inferences for 2899 queries when first names were given and 2167 when country data was also supplied (732 names were associated with countries unsupported by genderize).

Sensitivity and specificity are measurements of an algorithm's tendency to return correct answers instead of false positives (e.g., a man incorrectly gendered as a woman) or false negatives (e.g., a woman incorrectly gendered as a man). The closer these values are to 1, the smaller the chance that the algorithm will return the correlating false response. Accuracy is a composite measure of the algorithm's ability to differentiate the genders correctly. These measurements were calculated from the data sets (with and without country data supplied) at three different probability threshold cutoffs: the default genderize (0.5), a probability threshold of 0.85 (0.85), and a modified probability of 0.85, which factors in the number of instances returned (pmod0.85; pmod = probability x count + 2/(count + 4))(2, 3).

At the 0.5 threshold, the data set returned a sensitivity of 0.8943 and specificity of 0.9339 for an accuracy of 0.911, compared to a marginally higher accuracy of 0.9146 for the data set where country data were included (Table S1). Generally speaking, the accuracy increases as the threshold increases, with slight trade offs between sensitivity and specificity. For the purposes of our analysis, we opted to use the pmod0.85 threshold for our analysis (Table S1, in bold).

**Impact of geographic bias.** To understand the extent of geographic bias in our gender inferrment against regions and languages with gender-less naming conventions or that lack social media for incorporation into the genderize algorithm, we compared the number of names inferred without associated country data to when country data was also supplied. In our test data set, the top five

countries associated with names were the United States, Germany, the United Kingdom, France, and China. The countries with the highest proportion of un-inferred genders when country data were supplied are Cambodia, Iceland, Indonesia, Ireland, and Mexico, where the maximum number of names supplied ranged from 1 to 15. To determine the impact of each country towards the overall percentage of names whose genders were not inferred (27.1%), we found the difference between the percent of names un-inferred for each country and the overall percentage, multiplied by the proportion of observations from that country to the total observations and finally divided by the overall percentage of un-inferred names (Fig. S1A). The top five countries with the greatest impact on un-inferred names, and thus the countries receiving the most negative bias from genderize were Canada, China, Ireland, Belgium, and Sweden. These data suggest that there is likely some bias against countries with gender-neutral naming conventions (e.g., China), and indicates the stringency with which the algorithm applies gender to names that are accompanied by country data. For instance, strongly gendered names such as Peter and Pedro were not inferred a gender when associated with Canada.

We next applied the genderize algorithm at the pmod0.85 threshold to our journals data set and tested its validity on a small portion. All first names collected from our data set were submitted to genderize both with and without country data and only those with a pmod equivalent to or greater than 0.85 were retained. Next, the inferred genders were assigned to individuals as described in the methods. Given the relatively small number of editors and senior editors in our data set, the presenting gender (man/woman) of editors and senior editors in our data set was hand-validated using Google where possible. Of the 1072 editor names, 938 were inferred by our application of genderize for an accuracy of 0.9989, thus increasing our confidence in the gender inferences.

In our full data set, the five countries with the most individuals were the United States, China, Japan, France, and Germany. The countries with the highest proportion of un-inferred genders were Burundi, Chad, Kingman Reef, North Korea, and Maldives, where the maximum number of names supplied ranged from 1 to 4. Proportionally, fewer names in our full data set were inferred gender than in our validation data set (40% un-inferred versus 27.1% un-inferred, respectively). Since adjusting the workflow to infer the gender of names both with and without country data, the countries receiving the most negative bias from genderize were China, Japan, South Korea,

India, and Taiwan (Fig. S1B). These data indicate what we previously inferred, that the genderize algorithm has bias against countries with gender-neutral naming conventions.

Table S1. Sensitivity, specificity, and accuracy of genderize thresholds for data provided by Broderick and Casadevall (1). Bolded text denotes the accuracy of the threshold used in all further analyses. Also included are the percent of names not inferred a gender by the API (percent unknown) at each threshold.

| | First Names | | | Plus Country Data | | |
|---|---|---|---|---|---|---|
| Measure | p0.5 | p0.85 | pmod0.85 | p0.5 | p0.85 | pmod0.85 |
| Sensitivity | 0.8943 | 0.9516 | 0.971 | 0.9055 | 0.9471 | 0.9669 |
| Specificity | 0.9339 | 0.9593 | 0.972 | 0.9265 | 0.9553 | 0.9727 |
| Accuracy | 0.9110 | 0.9549 | **0.9714** | 0.9146 | 0.9507 | **0.9695** |
| Percent Unknown | 11.2000 | 22.6000 | 34.6 | 33.5000 | 40.3000 | 58.7 |

*Note:*
Bolded text denotes the accuracy of the threshold used in all further analyses

1. **Broderick NA**, **Casadevall A**. 2019. Gender inequalities among authors who contributed equally. eLife **8**:e36399. doi:10.7554/eLife.36399.

2. **Edwards HA**, **Schroeder J**, **Dugdale HL**. 2018. Gender differences in authorships are not associated with publication bias in an evolutionary journal. PLOS ONE **13**:e0201725. doi:10.1371/journal.pone.0201725.

3. **Holman L**, **Stuart-Fox D**, **Hauser CE**. 2018. The gender gap in science: How long until women are equally represented? PLOS Biology **16**:20. doi:10.1371/journal. pbio.2004956.
